# BCAR1 Protein Plays Important Roles in Carcinogenesis and Predicts Poor Prognosis in Non-Small-Cell Lung Cancer

**DOI:** 10.1371/journal.pone.0036124

**Published:** 2012-04-27

**Authors:** Wei Huang, Bo Deng, Ru-Wen Wang, Qun-You Tan, Yong He, Yao-Guang Jiang, Jing-Hai Zhou

**Affiliations:** Thoracic Surgery Department, Institute of Surgery Research, Daping Hospital, Third Military Medical University, Chongqing, People's Republic of China; The Moffitt Cancer Center & Research Institute, United States of America

## Abstract

**Objective:**

Our previous study suggested the potential clinical implications of BCAR1 in non-small-cell lung cancer (NSCLC) (Mol Diagn Ther. 2011. 15(1): 31–40). Herein, we aim to evaluate the predictive power of BCAR1 as a marker for poor prognosis in NSCLC cases, verify the carcinogenic roles of BCAR1 in the A549 lung adenocarcinoma cell line, and testify to the BCAR1/phospho-p38 axis.

**Methods:**

Between January 2006 and June 2010, there were a total of 182 patients with NSCLC (151 cases with available follow up data, and 31 cases lost to follow-up due to the invalid contact information). We inspected BCAR1, phospho-BCAR1(Tyr410), phospho-p38(Thr180/Tyr182) and p38 expression in NSCLC tissues and matched adjacent normal tissues by immunoblotting and IHC. After BCAR1 -RNA interference in A549 cells, we inspected the protein expression (BCAR1, phospho-BCAR1, phospho-p38 and p38) and performed cell biology experiments (cell growth, migration and cycle).

**Results:**

BCAR1 was overexpressed in NSCLC tissues (177/182) and cell lines (A549 and Calu-3). However, it was not detected in the normal adjacent tissue in 161 of the 182 cases. Higher BCAR1 levels were strongly associated with more poorly differentiated NSCLC and predicted poorer prognosis. BCAR1 knockdown caused cell growth arrest, cell migration inhibition and cell cycle arrest of A549 cells. Overexpression of BCAR1 was associated with activation of p38 in NSCLC cases, and BCAR1 knockdown caused reduction of phospho-p38 levels in A549 cells.

**Conclusion:**

Overexpression of BCAR1 is a predictor of poor prognosis in NSCLC and plays important carcinogenic roles in carcinogenesis, probably via activation of p38 MAPK. However, further investigations are required immediately.

## Introduction

Every year, there are 1.35 million new lung cancer cases in the world [1]. Worldwide, Lung cancer is the leading cause of cancer-related deaths [2]. Due to the intricate biological functions, prognosis of lung cancer remains very poor [1]. Hence, it is vital to unveil the biological functions of the disease for the sake of improving therapeutic efficacy [3].

Breast cancer anti-estrogen resistance 1 (BCAR1), also entitled p130cas, was one of the CAS protein (Crk-associated substrate) family members. It was originally identified as a cellular protein migrating at 130 kDa, and to be hyperphosphorylated in v-Crk and v-Src transformed cells [4], [5]. Initially, intensive studies were focused on the correlation between breast cancer and BCAR1 [6], [1], [7], [8]. For instance, Brinkman et al. [7] suggested BCAR1 overexpression in ZR-75-1 breast cancer cell line renders antiestrogen resistance to the cells. Furthermore, Dorssers et al. [8] reported that BCAR1 expression was inversely related to relapse-free survival and overall survival time of breast cancer.

Recently, our study suggested there is a clinical implication of BCAR1 in non-small-cell lung cancer (NSCLC) [9]. We investigated serum BCAR1 levels in 80 NSCLC cases and 80 healthy controls, respectively, by using a specific enzyme-linked immunosorbent assay (ELISA) [9]. Intriguingly, we found that serum BCAR1 levels were significantly higher in NSCLC than in the control group, increased gradually with the progression of tumor staging, and decreased after removal of the malignant lesions [9]. However, the oncogenic mechanisms of BCAR1 in NSCLC are still the enigmas.

Herein, we conducted the further investigations to evaluate the predictive power of BCAR1 as a biomarker for poor prognosis in NSCLC patients. And we verified the carcinogenic roles of BCAR1 via RNA interference (RNAi) in A549 lung adenocarcinoma cell line. Experiments in vivo and vitro demonstrated the closed correlation between BCAR1 expression and activation of p38 which is a crucial branch of the MAPK (mitogen-activated protein kinase) pathway [10], [11].

## Materials and Methods

### Patients

The study protocol was reviewed and approved by the Research Ethics Board in Daping hospital (Chongqing City, P.R.China) [reference no. TMMU-DPH/2006-012], and informed consent was written and obtained from all the patients.

In the study performed between January 2006 and June 2010, there were a total of 182 patients with NSCLC. Pulmonary neoplasm was diagnosed radiographically and confirmed by pathology. None of the patients had received treatment before enrollment in the study. The demographic and clinicopathological characteristics of patients are shown in table 1. All the patients underwent lobectomy and lymphadenectomy. One hundred and thirty five patients received postoperative adjuvant chemotherapy (Paclitaxel plus Nedaplatin). Postoperative follow-up was available in 151 cases by telephone or letter interview, and 31 cases lost to follow-up due to the invalid contact information.

**Table 1:** The patients’ clinical and pathological characteristics according to BCAR1 and p-p38 expression in NSCLC.

Characteristics	No.	BCAR1					p-p38				
		−	+	++	+++	*P*	−	+	++	+++	*P*
Group											
NSCLC	182	5	75	60	42	0.000#	101	36	32	13	0.000#
Normal tissue	182	156	26	0	0		148	32	2	0	
Age											
<60	78	3	31	30	14	0.507*	49	12	12	5	0.129*
≥60	104	2	44	30	28		52	24	20	8	
Gender											
Female	56	1	16	22	17	0.16*	31	11	7	7	0.792*
Male	126	4	59	38	25		70	25	25	6	
Histology											
squamous cell carcinoma	115	4	51	37	23	0.11*	68	20	20	7	0.242*
adenocarcinomas.	67	1	24	23	19		33	16	12	6	
Differentiation											
Well	63	0	37	18	8	0.010▵	37	11	11	4	0.665▵
Moderately	97	4	32	34	27		53	20	17	7	
Poorly	22	1	6	8	7		11	5	4	2	
TNM stage											
I	41	0	21	16	4	0.241▵	23	7	8	3	0.889▵
II	91	3	35	30	23		48	22	14	7	
III	47	2	17	13	15		28	7	9	3	
IV	3	0	2	1	0		2	0	1	0	
lymphonode status											
N0	128	4	54	40	30	0.711*	71	24	23	10	0.818*
N1–N3	54	1	21	20	12		30	12	9	3	
Tumor size											
<3 cm	101	3	48	26	24	0.206*	57	21	16	7	0.523*
≥3 cm	81	2	27	34	18		44	15	18	6	

Note:

# : *X* ^2^ test for the positive rate of staining (NSCLC vs Normal adjacent tissue);

* : Mann-whitney U test;

▵ : Kruskal-Wallis test.

### Cell Culture

A549 and Calu-3 lung adenocarcinoma cell lines were obtained from the American Type Culture Collection and cultured in RPMI1640/10% FBS, respectively.

### RNA Interference of BCAR1

Firstly, the following oligoribonucleotide pairs were used: 5′- CCGGGGTCGACAGTGGTGTGTATTTCAAGAGAATACACACCACTGTCGACCTTTTTTg-3′ and 5′-AATTCAAAAAAGGTCGACAGTGGTGTGTATTCTCTTGAAATACACACCACTGTCGACC-3′. Entire sequences were derived from the sequence of human BCAR1 mRNA. The oligonucleotides were obtained from Sunbio Medical Biotechnology CO., Ltd (Shanghai City, P.R.China). The complementary two strands (each at 20 µM) in 60 µl of annealing buffer (Sunbio Medical Biotechnology CO., Ltd, Shanghai City, P.R.China) were heated for 5 min at 95°C and then incubated for 1 h at room temperature. Thereafter, the GFP-tagged lentiviral vector pLVT351.LV for BCAR1-RNAi was constructed by inserting the annealing nucleotides into the Age I+EcoR I site of pMAGic 4.1 (Sunbio Medical Biotechnology CO., Ltd, Shanghai City, P.R.China).

Secondly, A549 cell line was plated at 2.3×10^5^ cells per well of 24-well cell culture plate and infected with lentivirus at a multiplicity of infection (MOI) of 10. Cells infected with pLVT351-L.V. and CMV-GFP-L.V.(blank lentiviral vector, pMAGic 4.1) was named as A549-BCAR1-RNAi and A549-negative control, respectively.

### Western Blotting analysis of BCAR1, phospho-BCAR1, phospho-p38 and p38 in NSCLC tissues and cells

NSCLC and matched adjacent normal tissues (at least 5 cm away from the primary tumors) were available from a total of sixty (including 35 adenocarcinomas and 25 squamous cell carcinomas) cases for western blotting. During the operations, the samples were collected by the technicians promptly following removals. Besides, we also prepared the cell lines including Calu-3, A549, A549-negative control and A549-BCAR1-RNAi.

Thereafter, lysates from tissues and cell lines were prepared in a RIPA buffer comprising 50 mM Tris–HCl pH 7.5, 150 mM NaCl, 1% Triton X-100, 0.1% SDS, 0.5% deoxycholic acid and 0.02% sodium azide. The protein concentrations were determined with a BCA Protein Assay Kit (Pierce, Rockford, IL).

Proteins were denatured at 95°C for 5 min, and 50 µg protein per lane was resolved by sodium dodecyl sulfate-polyacrylamide gel electrophoresis (SDS-PAGE) using 10% polyacrylamide gel. Proteins were blotted on polyvinylidene difluoride (PVDF) membranes (Thermo), which were then blocked with 5% skim milk for 1 h at room temperature. The proteins were immunoblotted using anti-BCAR1 antibody(BD Transduction Laboratories, USA, 1∶1000), anti-phospho-BCAR1 (Tyr410) antibody (Abcam, USA, 1∶1000), anti-p38 (BD Transduction Laboratories material, USA, 1∶1000) and anti-phospho-p38(Thr180/Tyr182) antibody (cell signaling Transduction Laboratories, USA, 1∶1000). An anti-β-actin(Sigma) or GAPDH (Sigma) antibody served as the control.

Gray scales of immunoblotting of NSCLC and matched adjacent normal tissues were quantitatively analyzed by using image acquisition and analysis software (Image Lab software, BIO-RAD, USA) according to the manufacturer's instructions.

### Tissue microarray construction and Immunohistochemical (IHC) assay of BCAR1, phospho-BCAR1, p38 and phospho-p38 in NSCLC tissues

The hematoxylin and eosin (H&E)-stained slides of all 182 cases were inspected. For each case, the pathologic diagnosis was identified on the H&E-stained slide and then circled. A corresponding slide of normal adjacent tissue was also marked. Tissue microarray was constructed according to the previously published protocols [9]. The marked slide was aligned with the surface of the corresponding donor block. Thereafter, the area was marked on the paraffin tissue blocks. From each specimen, tissue cores with a diameter of 1.5 mm were punched and then arrayed on a recipient paraffin block. Sections (4 mm) of these microarray blocks were cut and then used for the IHC analysis.

The sections were incubated with serum blocking solution and primary antibodies including anti-BCAR1 antibody (BD Transduction Laboratories, USA, 1∶100), anti-phospho-BCAR1 (Tyr410) antibody (Abcam, USA, 1∶100), anti-p38 antibody (BD Transduction Laboratories, USA, 1∶50) and anti-phospho-p38 (Thr180/Tyr182) antibody (cell signaling Transduction Laboratories, USA, 1∶10), biotinylated secondary antibody, and streptavidin-horseradish peroxidase. Diaminobenzidine solution was used as a chromogen. The slides were then counterstained in a hematoxylin solution. The IHC results on protein abundance were classified according to the percents of positive cells as follows: −, negative staining; +, weak position (<25%); ++, moderate position (<50%); +++, strong position (≥50%). “<25%" was classified as low expression, otherwise as high expression. Three observers carried out to score the slides and mean values were obtained finally.

### TDT-mediated dUTP nick end labeling assay (TUNEL) of NSCLC tissues

We conducted TUNEL assay in tissue sections of all the 182 cases. Apoptosis in the tumor was inspected with In Situ Cell Death Detection kit, POD (Roche, Germany). Tissue sections were deparaffinize in xylene, and hydrated through graded ethanol and pretreated with microwave antigen retrieval (citrate buffer, pH 7.5). Endogenous peroxidase was blocked by 0.3% H_2_O_2_ in methanol for 15 min. Thereafter, tissue sections were treated with 3% bovine serum albumin for 30 min and incubated with TUNEL reaction mixture (TDT : dUTP, 1∶20) for 45 min at 37°C. Tissue sections were combined with Converter-POD, followed by washing and DAB (Zhong Shong, China) color reaction. During the TUNEL procedure, sections were washed in phosphate buffer saline (PBS). Tissue sections were counterstained by hematoxylin, dehydrated through graded ethanol, cleared in xylene, and mounted. For negative controls, deionized water was used instead of TDT. Positive controls consisted of inflamed human tonsil. Cells were considered positive when intense brown reactivity was detected in the nuclei. Apoptotic index was calculated the percentage of nuclear positive stain in 1000 cells in five different sites of each section at a 400×magnification.

### Real-time RT-PCR

To evaluate the efficacy of RNAi of BCAR1, we performed quantitative real-time RT-PCR in A549-BCAR1-RNAi and A549-negative control cells. Either of the cells was seeded at a concentration of 1×10^5^cells/well in 6-well plates. After two days seeding, total RNA was extracted from cells using Trizol reagent (Invitrogen). First-strand cDNA was synthesized with M-MLV transcriptase (Promega) and oligo dT. Real-time PCR was performed using SYBR Green PCR master mix (TAKARA) and the ABI Prism 7000 sequence detection system (Applied Biosystems, Foster City, CA). PCR primers were used as followings: 5′-CAATGCCTCACTGCTCTT-3′ and 5′-GTAGTCATAGTCCTCCATC-3′. The specificity of detected signals was confirmed by a dissociation curve consisting of a single peak. All samples were run in duplicate in each experiment. Values were normalized by human β-actin.

### Cell Growth Assay

For evaluation of cell growth, A549-negative control and A549-BCAR1-RNAi cells was plated at 2.0×10^2^ cells per well in six-well plates, respectively. And either of the cells was inspected and photographed following Giemsa staining, eleven days after plating.

### Cell Migration Assay

For assessment of cell motility, chamber migration assays were conducted using a cell culture insert (8-µm pore size, 24-well format; Cell Invasion Assay Kit Cat. CHEMICON, No. ECM550). A549-negative control and A549-BCAR1-RNAi cells were seeded in duplicate at a density of 3.0×10^5^ cells/chamber. After 48 hours, cells which had not moved to the lower wells were removed from the upper face of the filters using cotton swabs, and cells that had moved to the lower surface of the filter were stained by using a Cell Invasion Assay Kit Cat. (CHEMICON, No. ECM550). Cell migration was quantified by visual counting after being photographed. Experiments were performed in duplicate. Mean values for three random fields were obtained for each well.

### Cell Cycle Assays

For flow cytometric determination of cell cycles, 2.0×10^6^ of A549-negative control cells and A549-BCAR1-RNAi cells were fixed in 70% ethanol and stained with propidium iodide, respectively. The stained cells were analyzed on a FACScan flow cytometer for relative cell cycles. Experiments were performed in duplicate.

### Cell Apoptosis Assays

We evaluated cell apoptosis using FACScan flow cytometer. 2.0×10^6^ of A549-negative control cells (48 h after transient transfection), A549-negative control cells (stable transfection), A549-BCAR1-RNAi cells (48 h after transient transfection) and A549-BCAR1-RNAi cells (stable transfection) were fixed in 70% ethanol and stained with propidium iodide, respectively. The stained cells were analyzed on a FACScan flow cytometer for relative cell apoptosis. Experiments were performed in duplicate.

### Data Analysis

The statistical analysis was performed using the Student's t-test, Mann-whitney U test, Kruskal-Wallis test, *X* ^2^ test and Spearman's rho, respectively. Death from any cause was included in the calculation of postoperative survival. The disease specific survival was calculated by the Kaplan-Meier method and analyzed by the log-rank test. Prognostic factors were examined by univariate and multivariate analyses using a Cox proportional hazards model. All of the aforesaid calculations were performed using SPSS Version 11.0 software for Windows (SPSS, Inc., Chicago, USA). A value of p<0.05 (two-sided) was considered statistically significant.

## Results

### BCAR1 is overexpressed in NSCLC tissues and cell lines

**Figure 1:**
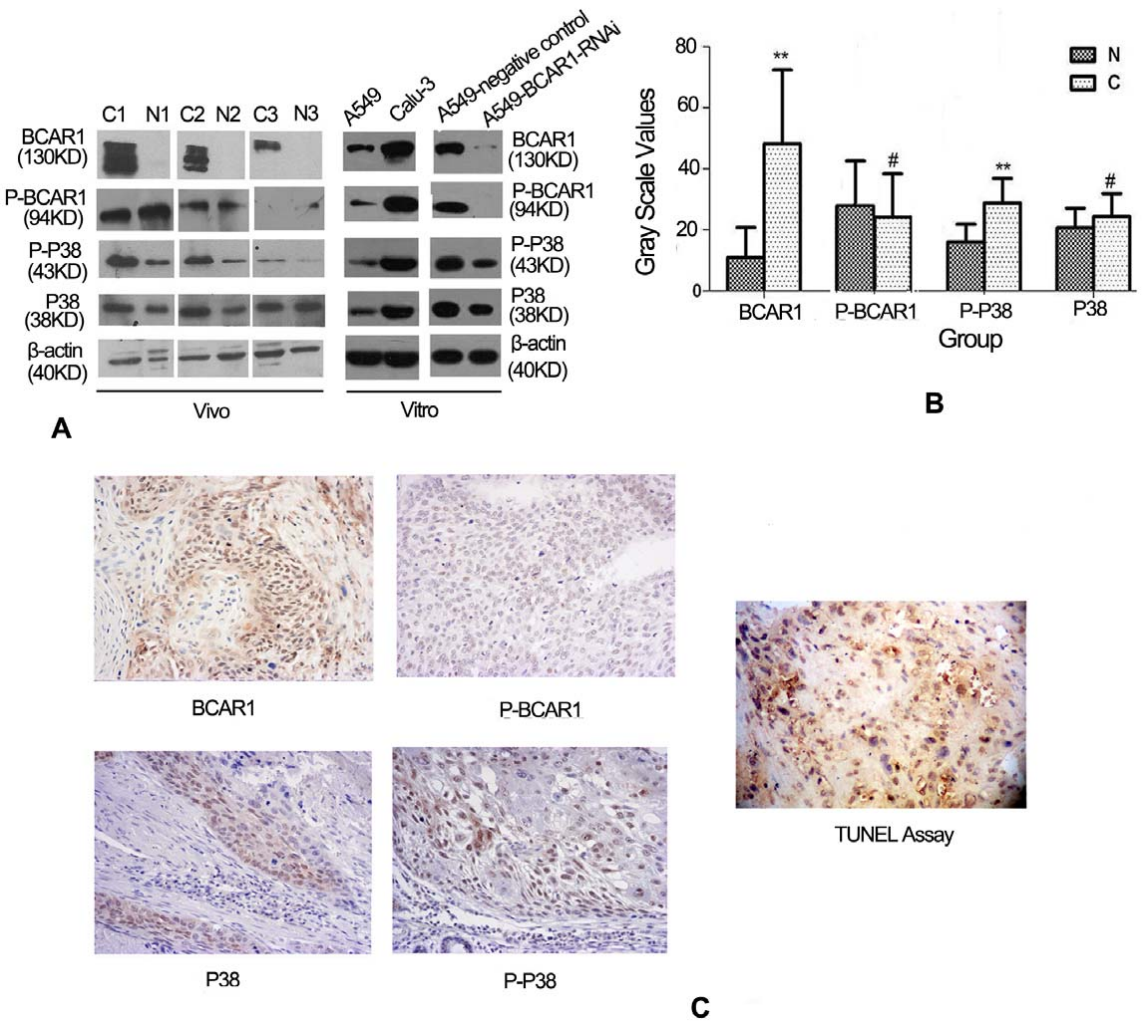
BCAR1, phospho-BCAR1, p38 and phospho-p38 expression in NSCLC tissues and cells. A: Immunoblotting indicated either BCAR1 or phospho-p38(Thr180/Tyr182) levels in three NSCLC tissues (C) were significantly higher than in the adjacent normal tissues (N). But phospho-BCAR1 (Tyr410) and p38 levels in NSCLC and the adjacent normal tissues were similar. BCAR1, phospho-BCAR1, p38 and phospho-p38 expressions were also detected in A549 and Calu-3 NSCLC cell line by using Immunoblotting assay. BCAR1 knockdown causes the appreciable reduction of phospho-BCAR1 and phospho-p38 levels in A549 cells. B: Gray scales analysis of immunoblotting also suggested BCAR1 and phospho-p38(Thr180/Tyr182) levels were significantly higher in NSCLC than in the normal adjacent tissue (48.18±24.7 vs 10.97±9.8,*P*<0.001; 16.03±5.8 vs 28.82±8.0, *P*<0.001). However, phospho-BCAR1 (Tyr410) and p38 had not the trend (20.72±6.4 vs 24.37±7.5, *P* = 0.22; 25.3±11.2 vs 27.8±15.2, *P* = 0.476). C: IHC suggested the expressions of BCAR1 (either in the nucleus, the cytoplasm), phospho- BCAR1 (prone to locate in the cytoplasm), phospho-p38 (prone to locate in the nucleus), p38 (prone to locate in the cytoplasm) and apoptotic bodies. Note: N (adjacent normal tissue); C (NSCLC tissue); ** (*P*<0.001); # (*P*>0.05).

BCAR1 expression was detected (either in the nucleus, the cytoplasm, or both) in 57 of the 60 NSCLC cases by using Immunoblotting (Figure 1a), and 177 of the 182 NSCLC cases by using IHC assay(Figure 1c), respectively. However, it was not detected in the normal adjacent tissue in 53 of the 60 cases by using Immunoblotting (Figure 1a), and 161 of the 182 cases by using IHC assay (Figure not shown), respectively. Analysis of gray scales of immunoblotting also suggested BCAR1 levels were significantly higher in NSCLC than in the normal adjacent tissue (48.2±24.7 vs 11.0±9.8, Student's t-test, *P*<0.001, Figure 1b).

However, phospho-BCAR1(Tyr410) was detected in cytoplasm in 29 of the 60 NSCLC by using Immunoblotting (Figure 1a), and 32 cases of the 182 NSCLC cases by using IHC assay(Figure 1c), respectively. However, it was also detected in the normal adjacent tissue in 30 of the 60 cases by using Immunoblotting (Figure 1a), and 34 of the 182 cases by using IHC assay (Figure not shown), respectively. Analysis of gray scales of immunoblotting suggested there was no appreciable difference of BCAR1 levels in between NSCLC and the normal adjacent tissue (25.3±11.2 vs 27.8±15.2, *P* = 0.476, Student's t-test, Figure 1b).

BCAR1 and phospho-BCAR1(Tyr410) was detected in A549 and Calu-3 NSCLC cells by using immunoblotting assay (Figure 1a).

### Higher BCAR1 levels are strongly correlated with more poorly differentiated tumors and predicts poorer prognosis

By using IHC assay in the 182 NSCLC cases, we found that higher BCAR1 levels were strongly correlated with more poorly differentiation. (Kruskal-Wallis test, *P* = 0.01; Table 1). However, there was no appreciable correlation between BCAR1 and other clinical-pathological characteristics including age, gender, histology, TNM stage, tumor size and lymphonode metastasis (Table 1). Despite BCAR1 expression was detected either in cytoplasm, nucleus, or both in NSCLC, there was no appreciable correlation between the protein location and clinical-pathological parameters (Data not shown). Besides, there was no significant correlation between phospho-BCAR1 and the clinical-pathological characteristics (Data not shown).

**Figure 2:**
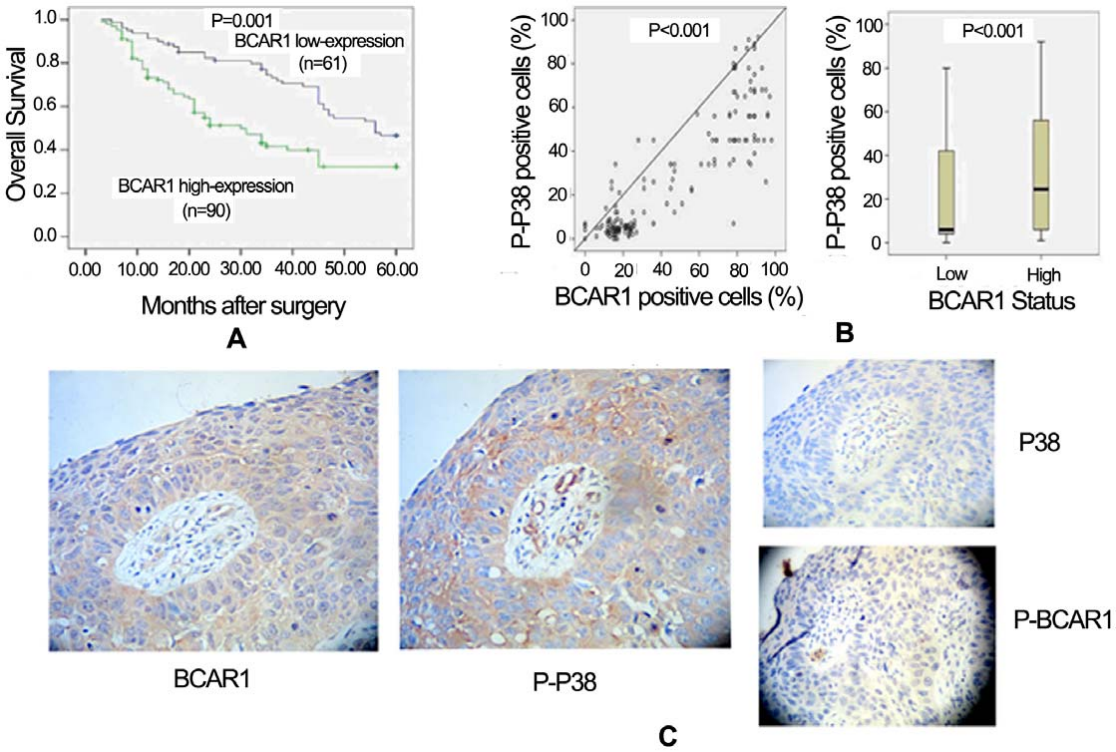
High levels of BCAR1 predict poorer prognosis, and are correlated to activation of p38. A: The survival rate of BCAR1 high-expressed group was significantly lower than of low-expressed group (*P* = 0.001). B: Phospho-p38 positive cells was significantly and positively correlated with percentages of BCAR1 positive cells, in the 182 NSCLC tissues (Spearman's rho, correlation coefficient = 0.811, p<0.001). And percentages of phospho-p38 positive cells in BCAR1 high-expressed tissues were significantly higher than in low-expressed tissues (Mann-whitney U test z = −3.689 *P*<0.001). C: The sequential sections were stained for BCAR1, phospho-BCAR1, phospho-p38 and p38, which demonstrated that the cells over-expressing BCAR1 also have a higher level of phospho-p38 (×200). Either phospho-BCAR1 or p38 was slightly positive.

The survival rate of BCAR1 high-expression group was significantly lower than of low-expression group (*P* = 0.001, Figure 2a). Multivariate analysis revealed that BCAR1 levels (hazard ratio 1.777, *P* = 0.028), lymphonode metastasis (hazard ratio 1.277, *P* = 0.040) and TNM stage (hazard ratio 1.298, *P* = 0.007) were significant and independent prognostic indicators for NSCLC cases (Table 2).

### BCAR1 knockdown causes cell growth arrest, cell migration inhibition and cell cycle arrest of A549 cells

**Figure 3:**
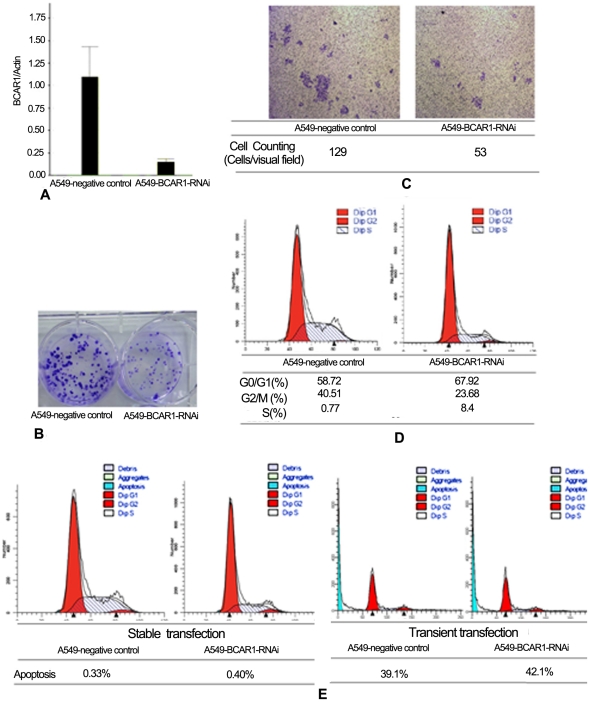
In A549 cells, BCAR1 knockdown caused cell growth arrest, cell migration inhibition, cell cycle arrest, but not apoptosis. A: At least an 80% reduction in mRNA of BCAR1 in A549-BCAR1-RNAi cells was confirmed by real-time RT-PCR. B: Colony forming units of A549-BCAR1-RNAi were detectably less than of A549-negative control cells, eleven days after plating. C: Cell migration assay suggested BCAR1 knockout prevented cell migration of A549-BCAR1-RNAi cells. D: Flow cytometry indicated BCAR1 knockout also caused cell cycle arrest of A549-BCAR1-RNAi cells. E: Flow cytometry indicated BCAR1 knockout did not increase apoptosis rate following either transient (39.1% vs 42.1%) or stable transfection (0.33% vs 0.4%) in A549 cells.

We established human NSCLC cancer A549 cells that stably expressed pLVT351-L.V. (A549-BCAR1-RNAi cells) and CMV-GFP-L.V. (A549-negative control cells) through use of a lentivirus system. At least an 80% reduction in mRNA levels of BCAR1 in A549-BCAR1-RNAi cells was confirmed by real-time RT-PCR (Figure 3a) and Western Blotting analysis (Figure 1a), respectively. Simultaneously, we can see the inhibition of phospho-BCAR1 along with BCAR1 knockdown (Figure 1a).

**Table 2:** Multivariate regression analysis in predicting the overall survival of NSCLC patients.

Variable	Hazard ratio	95% Cofidence interval	*P*-value
BCAR1 expressed levels	1.777	1.064–2.968	0.028
Phospho-BCAR1 levels	1.001	0.413–2.423	0.999
Age	0.960	0.643–1.433	0.843
Gender	0.778	0.493–1.225	0.278
Nodal status	1.277	1.018–1.668	0.040
Tumor size	1.113	0.748–1.655	0.597
Differentiation	1.449	0.753–2.794	0.267
TNM stage	1.298	1.068–1.588	0.007
Histology	1.103	0.689–1.543	0.882

Figure 3b suggested colony forming units of A549-BCAR1-RNAi cells were detectably less than of A549-negative control cells, eleven days after plating (Figure 3b). Cell migration assay suggested BCAR1 knockdown prevented cell migration of A549-BCAR1-RNAi cells (Figure 3c). And Flow cytometry indicated BCAR1 knockdown also caused cell cycle arrest of A549-BCAR1-RNAi cells (Figure 3d).

### BCAR1 is adversely correlated to apoptotic index in NSCLC tissues. However, BCAR1 knockdown can not increase apoptosis in A549 cells

Apoptotic bodies were detected in all the 182 NSCLC tissues (Figure 1c). Among the tissues, there was a significant and inverse correlation between BCAR1 and apoptotic index (Spearman's rho, correlation coefficient = −0.183; *P* = 0.013). Apoptotic index in BCAR1 high-expressed tissues was substantially lower than in BCAR1 low-expressed tissues (Student's t-test t = 2.312 *P* = 0.022).

However, in comparison with negative control cells, BCAR1 knockout did not increase apoptosis rate following either transient (39.1% vs 42.1%) or stable transfection (0.33% vs 0.4%) in A549 cells (Figure 3e).

### Overexpression of BCAR1 is correlated with activation of p38 in NSCLC cases and BCAR1 knockdown causes reduction of phospho-p38 in A549 cells

Phospho-p38 expression was detected in 31 of the 60 NSCLC cases by using immunoblotting (Figure 1a), and 91 of the 182 NSCLC cases (prone to locate in the nucleus) by using IHC assay (Figure 1c). Similar to the trend of BCAR1 levels, gray scales analysis revealed phospho-p38 levels were significantly higher in NSCLC than in the normal adjacent tissues (16.03±5.8 vs 28.82±8.0, *P*<0.001; Figure 1b). However, p38 had not the trend (20.72±6.4 vs 24.37±7.5, *P* = 0.22) (Figure 1b). Besides, IHC suggested positive rate of either BCAR1 or phospho-p38 was substantially higher in NSCLC than in normal tissue (*P*<0.001 and P<0.001, respectively) (table 1). However, p38 had not such trend (*P* = 0.62).

We found percentages of phospho-p38 positive cells was significantly and positively correlated with those of BCAR1 positive cells, in all the 182 NSCLC tissues (Spearman's rho, correlation coefficient = 0.811, p<0.001; Figure 2b). And percentages of phospho-p38 positive cells in BCAR1 high-expressed tissues were significantly higher than in BCAR1 low-expressed tissues (Mann-whitney U test z = −3.689 *P*<0.001; Figure 2b). However, there was not any correlation between phospho-BCAR1 and phospho-p38 levels (P = 0.892).

In an attempt to depict the strong correlation between BCAR1 positive and phospho-p38 positive cells, we presented the sequential sections stained in one case for BCAR1, phospho-BCAR1, phospho-p38 and p38, which demonstrated that the cells over-expressing BCAR1 also have a higher level of phospho-p38 (Figure 2c).

BCAR1 levels in Calu-3 cells were detectably higher compared with A549 cells (Figure 1a). Intriguingly, Figure 1a suggested phospho-p38 levels also had the same trend. Furthermore, BCAR1 knockdown also causes the appreciable reduction of phospho-p38 abundance in A549 cells (Figure 1a).

## Discussion

BCAR1 as an adapter protein localizes to chromosome 16q22-q23^7^, and mainly consists of four functional portions including an amino-terminal Src homology 3 (SH3) domain, a Src-binding domain (SBD), a large substrate domain (SD) and a helix-loop-helix domain (HLH) [12], [13]. BCAR1 locates ubiquitously in vitro and vivo [14], [15], [16], and is involved in various cellular processes including migration, chemotaxis, apoptosis, cell cycle, differentiation and so forth [17], [18].

Thus far, very few studies focused on the carcinogenesis of BCAR1 in lung cancer. Wei et al. [19] suggested that anchorage-independent phosphorylation of BCAR1 protected lung adenocarcinoma cells from anoikis. Recently, our study suggested that serum BCAR1 levels were significantly higher in NSCLC than in the control group, increased gradually with the progression of tumor staging, and decreased after removal of the malignant lesions [9]. As a result, we presumed a novel oncogenic role of BCAR1 in NSCLC. Herein, we aimed to verify the clinical implications of BCAR1 overexpression and NSCLC, and to elucidate the carcinogenetic mechanisms of BCAR1 in NSCLC. As expected, we found BCAR1 protein is especially abundant in NSCLC cells and tissues. And our studies in vivo and vitro showed the close correlation between BCAR1 expression and activation of p38 MAPK. Although prior studies have shown that tyrosine phosphorylation of BCAR1 can be critical for downstream signaling [20], our study indicated that phospho-BCAR1(Tyr410) was detected in only 34 of the 182 cases, and not correlated with clinical-pathological characteristics. Thus far, numerous sites of human BCAR1 phosphorylation are found as: tyrosine residues aa 12, 128, 165, 192, 222, 224, 234, 249, 267, 287, 306, 327, 362, 372, 387, 410, 653, 664, 666; serine residues aa 134, 139, 292, 437, 639; and threonine residues aa 269, 326, 385. We presume that some specific sites of phosphorylation are critical for the signal cascades of BCAR1 in NSCLC. Intriguingly, adjacent normal tissues show much higher levels of phospho-BCAR1 than of BCAR1 proteins (Figure 1a). We also confirmed the inhibition of phospho-BCAR1 along with BCAR1 knockdown in an attempt to verify the efficacy of this antibody. We presume that a special enzyme in lung tissues specifically decomposes Non-phospho-BCAR1, however, phospho-BCAR1 can survive. And all the abovementioned hypotheses deserve further more investigations.

Our experiments in vitro demonstrated that BCAR1 knockdown in A549 cells caused cell growth arrest, cell cycle arrest and cell migration inhibition. Although BCAR1 knockdown in A549 cells did not cause cell apoptosis, there was an appreciable and inverse correlation between BCAR1 and apoptotic index in NSCLC tissues. We do not know the reasons for discrepancy between NSCLC cell and tissue. Additionally, our study in vivo demonstrated that BCAR1 levels were significantly and inversely correlated with tumor differentiation, by either counting positive percents of cells (Kruskal-Wallis test, *P* = 0.010) or evaluating stained intensity (achromasy = 0, stramineous = 1, buffy = 2, brown = 3; Kruskal-Wallis test, *P* = 0.014). Kaplan-Meier curve also indicated that higher levels of BCAR1 predicted poorer prognosis in 151 cases with valid follow up data. All the aforementioned experiments suggested BCAR1 had a crucial role in carcinogenesis. Besides, BCAR1 is known as Src substrate, and Src inhibitor AZD0530 can also result in significant inhibition of cell migration and matrigel invasion in lung cancer cells [21], which potentially supports the carcinogenesis of Src/BCAR1 axis.

Using immunoblotting, Greenberg et al. [22] found that only activated p38 MAPK was consistently increased in NSCLC in comparison with normal tissue, suggesting an additional role for this pathway in malignant cell growth or transformation. Indeed, numerous studies had unveiled the carcinogenetic activities of p38, including stimulation of proliferation and migration [1], [23], [24]. Matsuda K et al. [23] found that EGF promotes proliferation via p38 MAPK signaling cascades in ASPC-1, PANC-1 and T3M4 pancreatic cancer cell lines, and p38 MAPK inhibitor SB203580 can inhibit EGF-stimulated mitogenesis. Besides, the p38 MAPK pathway has been implicated to play an important role in endothelial cell migration because inhibiting p38MAPK activity down-regulates VEGF-stimulated migration [24]. Recently, Wendt et al.'s study [25] demonstrated that increasing expression of either the full-length or just the carboxyl terminus of BCAR1 in mammary epithelial cells increased p38 activation. Intriguingly, in 182 NSCLC tissues, we found there was a close correlation between the expression of BCAR1 and phospho-p38. By evaluating stained intensity of phospho-p38 and BCAR1 (achromasy = 0, stramineous = 1, buffy = 2, brown = 3, “<2 scores" were classified as low expression, otherwise as high expression), we found the abundance of phospho-p38 was also significantly and positively correlated with that of BCAR1 (Spearman's rho, p<0.001). Besides, BCAR1 knockdown also caused the appreciable reduction of phospho-p38 levels in A549 cells. However, the underlying mechanisms deserve further investigations. Additionally, Figure 1a demonstrated BCAR1 knockdown slightly decreased expression of total p38 in A549 cells. Furthermore, adjacent normal tissues had low BCAR1, but not a decreased level of total p38. We think that the expression of total p38 is regulated by the other signal cascades except for BCAR1 in NSCLC.

Collectively, BCAR1 is associated with poor prognosis of NSCLC patients. BCAR1 knockdown experiments in A549 cells strongly supported the carcinogenesis of BCAR1 in NSCLC, probably via the activation of p38 MAPK. And we presume BCAR1 may be a potential therapeutic target for NSCLC. However, the further investigations are required immediately as: (i)Mechanistic link between BCAR1 and phospho-p38 should be studied; (ii) Stronger evidence should be provided to verify carcinogenesis of BCAR1 through activation of p38; (iii) The other key signaling molecules altered or activated by BCAR1, other than p38, should be evaluated.

## References

[1] Rooney C, Sethi T (2011). The epithelial cell and lung cancer: the link between chronic obstructive pulmonary disease and lung cancer.. Respiration.

[2] Ulahannan SV, Brahmer JR (2011). Antiangiogenic agents in combination with chemotherapy in patients with advanced non-small cell lung cancer.. Cancer Invest.

[3] Huang C, Liu D, Masuya D, Nakashima T, Kameyama K (2005). Clinical application of biological markers for treatments of resectable non-small-cell lung cancers.. Br J Cancer.

[4] Reynolds AB, Roesel DJ, Kanner SB, Parsons JT (1989). Transformation-specific tyrosine phosphorylation of a novel cellular protein in chicken cells expressing oncogenic variants of the avian cellular src gene.. Mol Cell Biol.

[5] Kanner SB, Reynolds AB, Parsons JT (1991). Tyrosine phosphorylation of a 120-kilodalton pp60src substrate upon epidermal growth factor and platelet-derived growth factor receptor stimulation and in polyomavirus middle-T-antigen-transformed cells.. Mol Cell Biol.

[6] Tikhmyanova N, Little JL, Golemis EA (2010). CAS proteins in normal and pathological cell growth control.. Cell Mol Life Sci.

[7] Brinkman A, der Flier Sv, Kok EM, Dorssers LC (2000). BCAR1, a human homologue of the adapter protein p130Cas, and antiestrogen resistance in breast cancer cells.. J Natl Cancer Inst.

[8] Dorssers LC, Grebenchtchikov N, Brinkman A, Look MP, van BSP (2004). The prognostic value of BCAR1 in patients with primary breast cancer.. Clin Cancer Res.

[9] Deng B, Huang W, Tan QY, Fan XQ, Jiang YG (2011). Breast cancer anti-estrogen resistance protein 1 (BCAR1/p130cas) in pulmonary disease tissue and serum.. Mol Diagn Ther.

[10] Cuadrado A, Nebreda AR (2010). Mechanisms and functions of p38 MAPK signalling.. Biochem J.

[11] Ji C, Ren F, Xu M (2010). Caspase-8 and p38MAPK in DATS-induced apoptosis of human CNE2 cells.. Braz J Med Biol Res.

[12] Kim W, Seok KY, Soo KJ, Shin NY, Hanks SK (2008). The integrin-coupled signaling adaptor p130Cas suppresses Smad3 function in transforming growth factor-beta signaling.. Mol Biol Cell.

[13] Chodniewicz D, Klemke RL (2004). Regulation of integrin-mediated cellular responses through assembly of a CAS/Crk scaffold.. Biochim Biophys Acta.

[14] Sakai R, Iwamatsu A, Hirano N, Ogawa S, Tanaka T (1994). A novel signaling molecule, p130, forms stable complexes in vivo with v-Crk and v-Src in a tyrosine phosphorylation-dependent manner.. EMBO J.

[15] Honda H, Oda H, Nakamoto T, Honda Z, Sakai R (1998). Cardiovascular anomaly, impaired actin bundling and resistance to Src-induced transformation in mice lacking p130Cas.. Nat Genet.

[16] Defilippi P, Di SP, Cabodi S (2006). p130Cas: a versatile scaffold in signaling networks.. Trends Cell Biol.

[17] Takino T, Tamura M, Miyamori H, Araki M, Matsumoto K (2003). Tyrosine phosphorylation of the CrkII adaptor protein modulates cell migration.. J Cell Sci.

[18] Kim W, Kook S, Kim DJ, Teodorof C, Song WK (2004). The 31-kDa caspase-generated cleavage product of p130cas functions as a transcriptional repressor of E2A in apoptotic cells.. J Biol Chem.

[19] Wei L, Yang Y, Zhang X, Yu Q (2002). Anchorage-independent phosphorylation of p130(Cas) protects lung adenocarcinoma cells from anoikis.. J Cell Biochem.

[20] Brabek J, Constancio SS, Siesser PF, Shin NY, Pozzi A (2005). Crk-associated substrate tyrosine phosphorylation sites are critical for invasionand metastasis of SRC-transformed cells.. Mol Cancer Res.

[21] Purnell PR, Mack PC, Tepper CG, Evans CP, Green TP (2009). The Src inhibitor AZD0530 blocks invasion and may act as a radiosensitizer inlung cancer cells.. J Thorac Oncol.

[22] Greenberg AK, Basu S, Hu J, Yie TA, Tchou-Wong KM (2002). Selective p38 activation in human non-small cell lung cancer.. Am J Respir Cell Mol Biol.

[23] Matsuda K, Idezawa T, You XJ, Kothari NH, Fan H (2002). Multiple mitogenic pathways in pancreatic cancer cells are blocked by a truncated epidermal growth factor receptor.. Cancer Res.

[24] Yu J, Bian D, Mahanivong C, Cheng RK, Zhou W (2004). p38 Mitogen-activated protein kinase regulation of endothelial cell migration depends on urokinase plasminogen activator expression.. J Biol Chem.

[25] Wendt MK, Smith JA, Schiemann WP (2009). p130Cas is required for mammary tumor growth and transforming growth factor-beta-mediated metastasis through regulation of Smad2/3 activity.. J Biol Chem.

