# Correction: BCAR1 Protein Plays Important Roles in Carcinogenesis and Predicts Poor Prognosis in Non-Small-Cell Lung Cancer

**DOI:** 10.1371/annotation/384d93a8-eef6-406b-8903-c7009061b496

**Published:** 2012-08-15

**Authors:** Wei Huang, Bo Deng, Ru-Wen Wang, Qun-You Tan, Yong He, Yao-Guang Jiang, Jing-Hai Zhou

There is an error in the Funding section. The correct funding information is as follows: The work was supported by the National Natural Science Foundation of China (NSFC) (No. 81101782), NSF project CQ CSTC (No. CSTC2011BB5020), and NSF Third Military Medical University (No. 2010XQN36).

Also, it should be noted that the date found in the footer of the downloadable PDF version of this article is incorrect. The correct date of publication for this article is April 2012, as stated in the Abstract and citation. 

